# Microbiota-dependent expansion of testicular IL-17-producing Vγ6^+^ γδ T cells upon puberty promotes local tissue immune surveillance

**DOI:** 10.1038/s41385-020-0330-6

**Published:** 2020-07-30

**Authors:** Anneke Wilharm, Helena C. Brigas, Inga Sandrock, Miguel Ribeiro, Tiago Amado, Annika Reinhardt, Abdi Demera, Lisa Hoenicke, Till Strowig, Tânia Carvalho, Immo Prinz, Julie C. Ribot

**Affiliations:** 1 Institute of Immunology, Hannover Medical School, Hannover, Germany; 2 Instituto de Medicina Molecular, Faculdade de Medicina, Universidade de Lisboa, Lisboa, Portugal; 3 Department of Microbial Immune Regulation, Helmholtz Center for Infection Research, Braunschweig, Germany

## Abstract

γδT cells represent the majority of lymphocytes in several mucosal tissues where they contribute to tissue homoeostasis, microbial defence and wound repair. Here we characterise a population of interleukin (IL) 17-producing γδ (γδ17) T cells that seed the testis of naive C57BL/6 mice, expand at puberty and persist throughout adulthood. We show that this population is foetal-derived and displays a T-cell receptor (TCR) repertoire highly biased towards Vγ6-containing rearrangements. These γδ17 cells were the major source of IL-17 in the testis, whereas αβ T cells mostly provided interferon (IFN)-γ in situ. Importantly, testicular γδ17 cell homoeostasis was strongly dependent on the microbiota and Toll-like receptor (TLR4)/IL-1α/IL-23 signalling. We further found that γδ17 cells contributed to tissue surveillance in a model of experimental orchitis induced by intra-testicular inoculation of *Listeria monocytogenes*, as *Tcrδ*^−*/−*^ and *Il17*^*−/−*^ infected mice displayed higher bacterial loads than wild-type (WT) controls and died 3 days after infection. Altogether, this study identified a previously unappreciated foetal-derived γδ17 cell subset that infiltrates the testis at steady state, expands upon puberty and plays a crucial role in local tissue immune surveillance.

## Introduction

The male reproductive system is composed of a pair of testes, accessory glands, such as the seminal vesicles and the prostate and a series of ducts that serve to transport spermatozoa to the female reproductive tract. Whereas accessory glands secrete products of the seminal fluid that enable sperm viability and motility, the testis plays a central role as a unique environment where spermatogenesis occurs. This process is driven by Sertoli cells, integrated components of the seminiferous tubules that warrant an environment, in which germ cells can progress towards the mature stage of the spermatogenic cycle. On the other hand, Leydig cells secrete testosterone, which acts on the Sertoli and peritubular cells to ensure the stability of this environment as well as the formation of testicular interstitial fluid.^1^

For the past four decades, the testis has been regarded as an immune-privileged organ where germ cell antigens are protected from potential autoimmune responses.^1^ However, the notion of “immune privilege” needs to be revisited to acknowledge a physiological role for resident immune cell populations in the interstitial spaces of the testes. For example, it was shown that steady-state interactions between the immune system and meiotic germ cell antigens contribute to systemic tolerance.^2^ Moreover, secretion of anti-inflammatory cytokines by resident macrophages regulates the homoeostasis of the testicular immunosuppressive microenvironment.^3^ In addition, resident macrophages were reported to impact on steroidogenesis by regulating Leydig cell development and function.^4^ By highlighting a physiological role for immune cells present in male reproductive organs at steady state, these data provide important cues to our knowledge about male infertility.

Naturally, immune populations also provide a key line of defence in the testes against pathogenic bacteria, namely in response to *Escherichia coli (E. coli)*, *Chlamydia* or *Listeria monocytogenes* (*L. monocytogenes*).^5^ Thus, we hypothesised the existence of critical immune mechanisms within the testis that would keep pathogens at bay and ensure reproduction.

γδ T cells only represent a minority among all lymphocytes in blood or secondary lymphoid tissues, but are highly enriched in mucosal tissues.^6,7^ There, they play crucial roles in mucosal immunity by acting as a first line of defence against several pathogens and tumours, in particular by local production of inflammatory cytokines such as interleukin (IL)-17 and interferon (IFN)-γ.^7^

The role of γδ T cells has been well characterised in the female reproductive tract, namely in the uterus, where they display a Vγ6-biased T-cell receptor (TCR) repertoire at steady state, and expand in the placenta and uterine decidua upon pregnancy.^8^ Interestingly, it has been proposed that seminal plasma can induce IL-17 production by uterine γδ T cells, promoting local inflammation important for embryo implantation.^9^ Furthermore, they provide protection against viral infection.^10^ In contrast, γδ T cells from male genitourinary organs remain poorly characterised. Whereas a Vγ6 population has been shown to be recruited during bacterial infection in the testis,^11^ and to impact on autoimmunity in a model of orchitis,^12^ the mechanisms controlling this response have not been described. Furthermore, while their presence has been associated with infertility disorders in humans,^13^ a potential impact of γδ T cells on steady-state testicular physiology remains to be elucidated.

By combining microscopy and flow-cytometry analyses, and employing a series of genetically modified specific pathogen-free (SPF), as well as germ-free (GF) mice, this study provides a comprehensive characterisation of γδ T cells in the testis at steady state and during inflammation. We demonstrate that γδ T cells are highly biased towards a foetal-derived TCR repertoire dominated by gamma-chain variable region (Vγ) 6 and strongly expand in the interstitial space of healthy testis during puberty. Furthermore, we show that testicular γδ T cells account for almost all local IL-17 producers at steady state, and that their homoeostasis relies on the symbiotic microbiome and on the Toll-like receptor (TLR)4/IL-1α/IL-23 signalling axis. While they seemingly do not impact on steady-state spermatogenesis and testosterone production, we document an important role of γδ17 cells in testicular immune surveillance upon *L. monocytogenes* infection. As bacterial infections and associated inflammation within male reproductive organs can lead to orchitis and associated reproductive disorders,^14^ we believe that our study provides cues on protective immune mechanisms that may be exploited for new immune-mediated strategies against male infertility.

## Results

### Testicular γδ T cells display a typical phenotype biased for IL-17 production

**Fig. 1:**
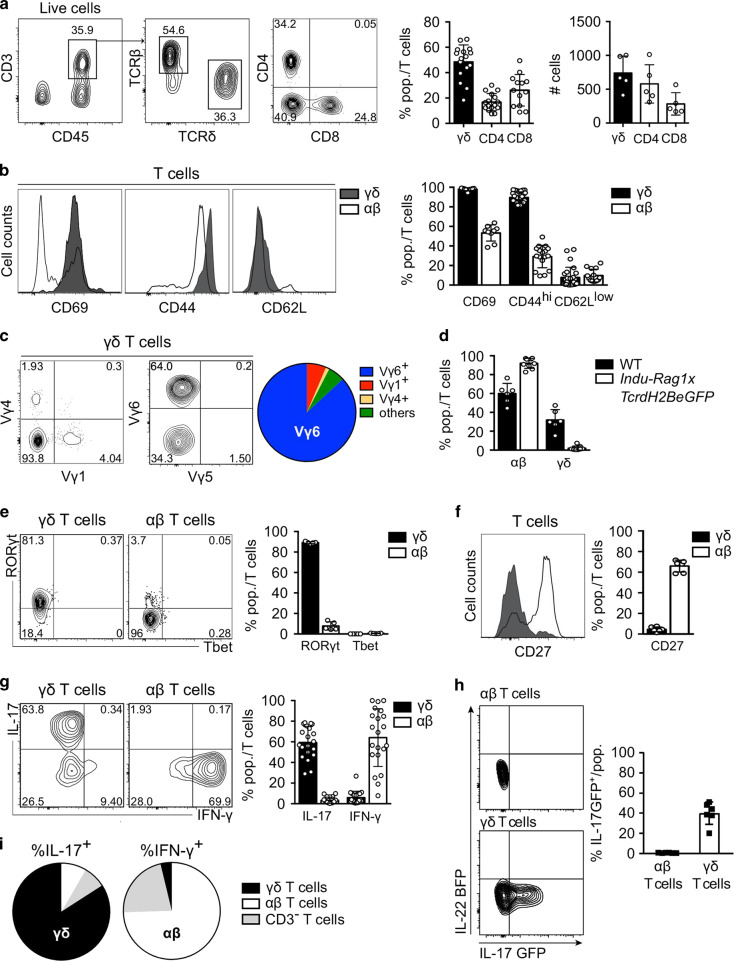
Testicular γδ T cells display a typical phenotype biased for IL-17 production. **a** Representative contour plots depicting γδ (middle) and CD4 and CD8 αβ (right) T cells gated on CD3^+^CD45^+^ cells (left) in testes of C57BL/6 mice (8–12 weeks old). Scatter plot shows frequencies and absolute numbers of γδ, CD4 and CD8 T cells among CD45^+^CD3^+^ cells (*n* = 5–16, two to five independent experiments). **b** Representative histogram of CD69, CD44 and CD62L expression of γδ (dark grey) and αβ (white) T cells. Scatter plot displays frequencies of indicated cell populations among γδ (black) and αβ (white) T cells (*n* = 8–17, two to five independent experiments). **c** Representative contour plot and pie chart depicting mean frequencies of Vγ1 + , Vγ4^+^, Vγ5^+^ and Vγ6^+^ γδ T cells in testes (*n* = 6–12, three independent experiments). **d** Scatter plot shows frequencies of αβ and γδ T cells in testes of WT (black) and *Indu-Rag1×TcrdH2BeGFP* (white) mice (*n* = 5–9, three independent experiments). **e** Representative contour plot and scatter plot of RORγt and T-bet expression in testicular γδ (left, black) and αβ (right, white) T cells (*n* = 5, one independent experiment). **f** Representative histogram of CD27 expression on γδ (grey) and αβ (white) T cells. Scatter plot with frequencies of CD27^+^ cells among γδ (black) and αβ (white) T cells (*n* = 6, two independent experiments). **g** Representative contour plot and scatter plot of IL-17 versus IFN-γ expression in testicular γδ (left, black) and αβ (right, white) T cells (*n* = 20–21, five independent experiments) after ex vivo stimulation of testicular lymphocytes with PMA and ionomycin. **h** Representative contour plot and scatter plot of IL-17 versus IL-22 expression in IL-17GFP/IL-22-BFP reporter mice, without prior stimulation by PMA and ionomycin (*n* = 3–6). **i** Pie chart depicting indicated immune cell subsets contributing to IL-17 (left) or IFN-γ (right) production in the testis after ex vivo stimulation by PMA and ionomycin (*n* = 12, three independent experiments). Data are represented as mean ± SD.

To characterise γδ T cells from the male reproductive tract, we analysed their distribution and phenotype in naive C57BL/6 mice by flow cytometry. We found that γδ T cells represented 50% of total CD3^+^ T cells in testis (Fig. 1a), while they were less frequent in prostate and seminal vesicle (SV) (Supplementary Fig. S1A). Testicular γδ T cells displayed a homogeneous activated CD69^+^CD44^hi^CD62L^low^ profile of tissue-resident effector T cells, while conventional αβ T cells were less activated in the testis (Fig. 1b). Importantly, the γδ TCR repertoire was mostly restricted to the usage Vγ6 (Fig. 1c). This was in sharp contrast to γδ T cells from the SV and prostate that comprised diverse Vγ subsets, and of which only 20% were activated CD44^hi^ cells (Supplementary Fig. S1B, C). The Vγ6 chain usually pairs with Vδ1 to form an invariant TCR in distinct foetal thymus-derived γδ T cells reported to colonise various non-lymphoid tissue in the perinatal period of life.^15^ To further confirm that embryonic thymus-derived Vγ6^+^ γδ T cells populate the testis, we analysed *Indu-Rag1×TcrdH2BeGFP* mice. Treating these mice with tamoxifen induces the expression of the Rag1 enzyme and thereby the maturation of B and T cells in adult organisms including γδ T cells. In addition, in *Indu-Rag1×TcrdH2BeGFP* mice, induced γδ T cells express histone-bound eGFP. *Indu-Rag1×TcrdH2BeGFP* mice not treated with tamoxifen lack γδ T cells.^16^ Hence, consistent with their embryonic origin and contrary to their αβ T-cell counterparts, Vγ6^+^ γδ T cells could not be reconstituted in the testis of *Indu-Rag1×TcrdH2BeGFP* mice after tamoxifen-mediated induction of Rag1 expression (Fig. 1d). In line with their Vγ6^+^ phenotype, testicular γδ T cells exhibited a typical signature of bona fide IL-17 producers,^17^ namely expressing the master transcription factor RORγt (Fig. 1e) while lacking CD27 (Fig. 1f). In contrast, αβ T cells expressed neither RORγt nor T-bet, but CD27, emphasising their naive phenotype within the testis (Fig. 1e, f). Importantly, the majority of testicular γδ T cells expressed IL-17, but not IFN-γ, after ex vivo stimulation with PMA and ionomycin (Fig. 1g), whereas γδ T cells from reproductive accessory glands produced equally IFN-γ and IL-17 (Supplementary Fig. S1D). Most importantly, we confirmed the testicular γδ T-cell phenotype using an IL-17-GFP/IL-22-BFP reporter mouse model^18^ (Fig. 1h), which allowed the detection of IL-17 producers in steady state while bypassing the need for PMA/ionomycin restimulation. Of note, the expression of IL-22 could neither be detected in the reporter mice (Fig. 1h) nor with a classical intracellular staining after PMA/ionomycin restimulation (data not shown).

We also assessed the contribution of γδ T cells to the entire cytokine production of ex vivo-stimulated lymphocytes from testes and accessory glands. In all cases, we observed a clear discrimination of function, since γδ T cells were the main producers of IL-17, whereas IFN-γ was mostly expressed by αβ T cells (Fig. 1i). Given their recently described impact on organ physiology,^19–21^ we decided to further focus on studying the homoeostasis and potential functions of Vγ6^+^ γδ17 infiltrating the testis.

### Vγ6^+^ IL-17^+^ γδ T cells accumulate specifically in the testis upon puberty

To investigate whether changes associated with physiological maturation of the reproductive tract could impact on γδ T-cell homoeostasis in the testis, we next characterised this population before and after puberty. We analysed testes of pre- (3–5-week-old) and post-pubertal (7–12-week-old) mice, as recommended by Jackson Laboratories.

**Fig. 2:**
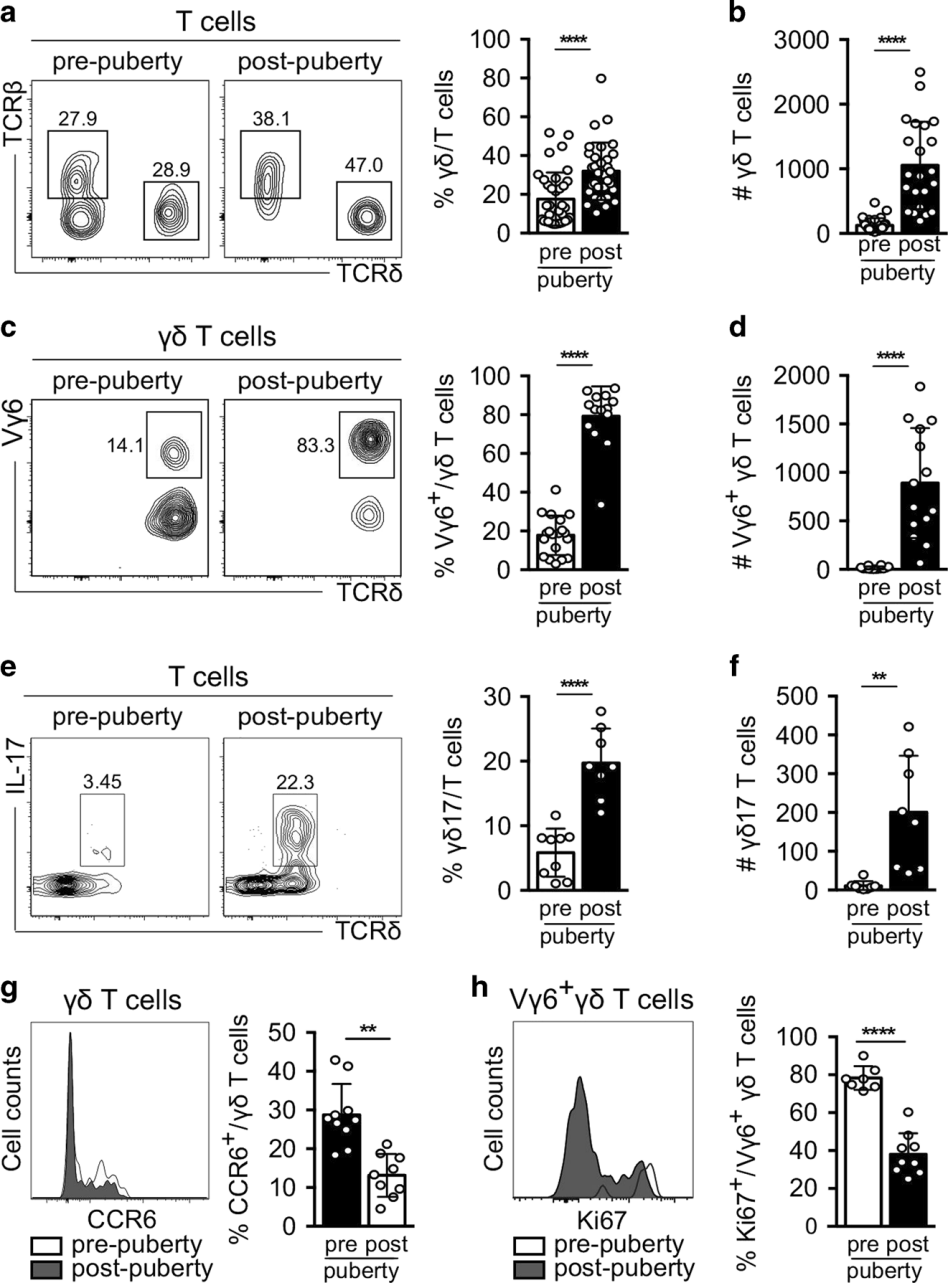
Vγ6^+^ IL-17^+^ γδ T cells accumulate specifically in the testis during puberty. **a** Representative contour plots depicting testicular γδ and αβ T cells gated on CD3^+^CD45^+^ cells before puberty (3–5-week-old (wo) mice) and post puberty (7–12-week-old mice). Scatter plot shows frequencies of γδ T cells among CD3^+^CD45^+^ cells in pre-pubertal (white) and post-pubertal (black) mice (*n* = 32–36, seven independent experiments). **b** Number (#) of γδ T cells in pre- (white) and post-pubertal (black) mice (*n* = 22–27, five independent experiments). **c** Representative contour plots depicting Vγ6^+^ γδ T cells before and after puberty. Scatter plot shows frequencies of Vγ6^+^ γδ T cells among lymphocytes in pre-pubertal (white) and post-pubertal (black) mice (*n* = 14–19, three independent experiments). **d** Number of Vγ6^+^ γδ T cells in pre- (white) and post-pubertal (black) mice (*n* = 14, three independent experiments). **e** Representative contour plots depicting IL-17-producing γδ T (γδ17) cells before and after puberty. Scatter plot shows frequencies of γδ17 cells in pre-pubertal (white) and post-pubertal (black) mice (*n* = 8–9, two independent experiments). **f** Number of γδ17 cells in pre- (white) and post-pubertal (black) mice (*n* = 8, two independent experiments). **g** Representative histogram of CCR6 expression of γδ in pre- (white) and post-pubertal (dark grey) mice. Scatter plot shows frequencies of CCR6^+^ γδ in pre-pubertal (white) and post-pubertal (black) mice (*n* = 8–9, two independent experiments). **h** Representative histogram of Ki67 expression of Vγ6^+^ γδ T cells in pre- (white) and post-pubertal (dark grey) mice (*n* = 7–9, three independent experiments). Data are represented as mean ± SD as evaluated by unpaired Student’s *t* test, **P < 0.01, ***P < 0.001, ****P < 0.0001.

Our data show that γδ T cells colonise the testis before puberty, as depicted by the small but sizeable population of testicular γδ T cells observed in 3–5-week-old mice, but expand drastically during puberty (Fig. 2a, b). The observed 12-fold increase in cell numbers was mainly due to the Vγ6^+^ subset (Fig. 2c, d) and IL-17-producing γδ T cells (Fig. 2e, f). While γδ T-cell infiltration of the testes may be partially driven by CCR6 (Fig. 2g), as previously reported,^22^ our data also suggested that the maturing testicular microenvironment specifically triggers the proliferation of Vγ6^+^ T cells during the onset of puberty, as this subset exhibited enhanced proliferative marks when stained for Ki67 (Fig. 2h).

### Microbiota, TLR4 and IL-23 signalling drives the accumulation of γδ17 cells in the testis

**Fig. 3:**
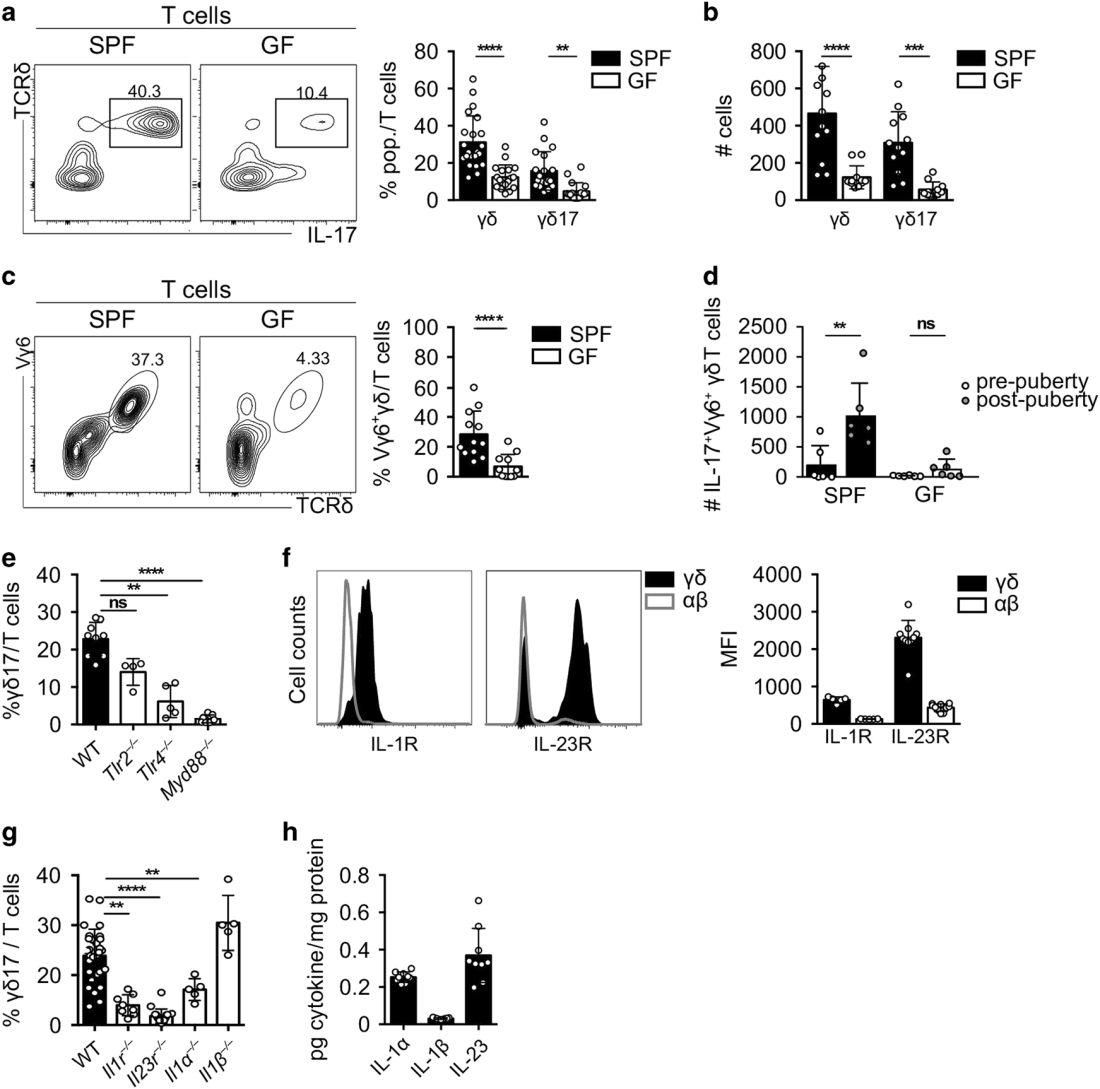
Accumulation of γδ T cells in the testis is dependent on microbiota, IL-23 and TLR4 signalling. **a** Representative contour plots depicting IL-17-producing γδ T cells gated on CD3^+^CD45^+^ cells in testes of specific pathogen-free (SPF) (left) and germ-free (GF) (right) mice. Scatter plot shows frequencies of γδ and γδ17 T cells among lymphocytes in testes of SPF (black) and GF (white) mice (*n* = 18–22, five independent experiments). **b** Number of testicular γδ and γδ17 cells of SPF (black) and GF (white) mice (*n* = 11–12, three independent experiments). **c** Representative contour plots depicting testicular Vγ6^+^ γδ T cells gated on CD3^+^CD45^+^ cells of SPF and GF mice. Scatter plot displays frequencies of Vγ6^+^ γδ T cells among all T cells in testes of SPF (black) and GF (white) mice (*n* = 11–14, four independent experiments). **d** Number of Vγ6^+^ γδ T cells from SPF and GF in pre- and post-pubertal mice (*n* = 6, two independent experiments) (*n* = 7–8, three independent experiments). **e** Scatter plot shows frequencies of γδ17 T cells among lymphocytes of WT (black), *Tlr2*^*−/−*^, *Tlr4*^*−/−*^ and *Myd88*^*−/−*^ (white) mice (*n* = 4–9, one to two independent experiments). **f** Representative histogram of γδ (dark grey) and αβ (white) T cells expressing IL-1 receptor (R) in WT mice (left) and IL-23R in *Il23r*^*gfp/gfp*^ mice (right) and scatter plot with mean fluorescence intensity (MFI) (*n* = 5–10, one to three independent experiments). **g** Scatter plot depicts frequencies of γδ17 among all T cells in testes of WT (black), *Il1r*^*−/−*^, *Il23r*^*−/−*^, *Il1α*^*−/−*^ and *Il1β*^−*/−*^ (white) (*n* = 5–29, one to three independent experiments). **h** Scatter plot displays picogram (pg) per mg protein of IL-1α, IL-1β and IL-23 in the testis (*n* = 9, two independent experiments). Data are represented as mean ± SD as evaluated by Kruskal–Wallis test followed by Dunn’s multiple-comparison test or one-way ANOVA followed by Holm–Sidak’s multiple-comparison test. **P < 0.01, ***P < 0.001, ****P < 0.0001.

We next investigated the molecular cues underlying the accumulation of testicular γδ T cells upon puberty. The maturation of the reproductive tract generally associates with important environmental changes, including a boost of steroidogenesis^23^ and a bidirectional crosstalk between the male endocrine system and the symbiotic microbiome established during puberty.^24,25^ Given that the microbiota can promote the differentiation of IL-17-producing CD4^+^ cells in the gut^26^ and can control the migration and homoeostasis of γδ17 cells to different tissues,^27–29^ we hypothesise that commensal bacteria could also influence testicular γδ17 cell accumulation at puberty. Thus, we compared the immune populations within the testis from adult mice hosted in GF versus SPF conditions. Adult GF mice exhibited a significant reduction in γδ T-cell frequencies and absolute numbers (Fig. 3a, b). Among total testicular γδ T cells, the IL-17-producing Vγ6^+^ subset was selectively affected in GF compared with SPF mice (Fig. 3c, d). By contrast, the production of testicular IFN-γ was not altered by the absence of symbiotic bacteria (Supplementary Fig. S2). Importantly, an increase in IL-17-producing Vγ6^+^ γδ T cells as observed in SPF mice post puberty did not similarly occur in GF mice, confirming a microbiota-dependent expansion of testicular γδ17 cells at puberty (Fig. 3d).

Commensal microbiota and their metabolites can act as pathogen-associated molecule patterns (PAMPs) to trigger TLR- signalling.^30^ Importantly, TLR4- and TLR2-stimulated myeloid cells promote γδ17 cell proliferation in an inflammatory set-up through the production of IL-1β and IL-23.^31,32^ Therefore, we asked whether γδ17 cell frequencies in the testis were influenced by TLR signalling and downstream cytokines, and analysed mice deficient for these candidates. Interestingly, we observed that steady-state γδ17 cell homoeostasis in the testes mainly relied on TLR4- but not TLR2 signals, as this subset was significantly reduced in mice deficient (^−*/−*^) for TLR4, whereas it was not significantly affected in *Tlr2*^−*/−*^ mice (Fig. 3e). Of note, TLR4 in the testis was mainly expressed by dendritic cells (Supplementary Fig. S3A). Consistently, the percentage of γδ17 cells was also decreased in *Myd88*^*−/−*^ mice, a key adaptor protein downstream of most TLR-signalling pathways (Fig. 3e).

We next anticipated that TLR4 triggering would promote the production of IL-1 and IL-23, which would in turn be responsible for γδ17 cell accumulation in the testis. In line with this, we observed that testicular γδ T cells constitutively expressed the receptors for IL-1 and IL-23, in sharp contrast with their αβ counterpart (Fig. 3f). Importantly, *Il1r*^*−/−*^ or *Il23r*^*−/−*^ mice recapitulated the reduction of γδ17 cell percentages observed in *Tlr4*^*−/−*^ animals, pointing at a crucial and non-redundant function of these cytokines for steady-state testicular γδ T-cell homoeostasis (Fig. 3g). Interestingly, IL-1α rather than IL-1β was seemingly required in this process (Fig. 3g, h). Altogether, these data indicate that testicular γδ17 cells are regulated by IL-1α/IL-23 and the TLR4/MyD88 signalling pathways potentially triggered by symbiotic microbial cues.

### γδ T cells do not influence steady-state testicular physiology

**Fig. 4:**
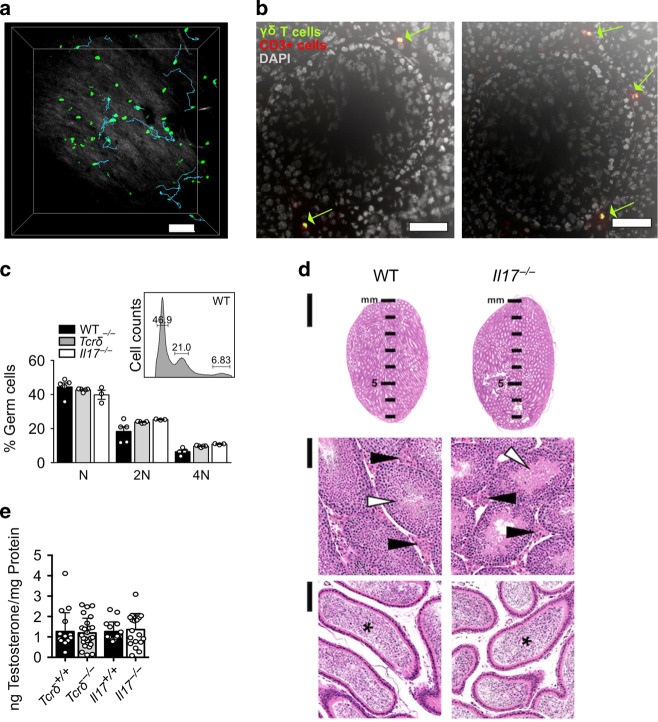
γδ T cells do not impact on testicular steady-state physiology. **a** Two-photon microscopy of the testis of adult *TcrdH2BeGFP* mice demonstrating γδ T cells (green) and collagen structures (white–grey). Using IMARIS software, motile γδ T cells were tracked (cyan lines). Scale bar represents 70 μM (*n* = 6 movies). **b** Representative immunofluorescence staining of cross sections from the testis of adult *TcrdH2BeGFP* mice against CD3 (red) and DAPI (white) for nuclear visualisation. Scale bars represent 50 μM (*n* = 4). **c** Representative histogram of germ cells in a WT mouse. Plot shows frequencies of germ cells of WT, *Tcrδ*^*−/−*^ and *Il17*^*−/−*^ mice (*n* = 3–5, one to two independent experiments). **d** Representative microphotographs of the testis of WT and *Il17*^−*/−*^ mice. Organs are alike in aspect, proportion and volume, with similar contribution of seminiferous tubules (white arrowhead), Leydig cells in the stromal compartment (black arrowhead) and with numerous spermatozoa in the lumen of the epididymis (asterisk). Haematoxylin and eosin staining. Original magnification ×1.25 (top column, bar, 2 mm) and ×20 (middle and lower columns, bar, 100 µm) (*n* = 3). **e** Scatter plot displays ng of testosterone/mg of protein in *Tcrδ*^−*/−*^ and *Il17*^*−/*−^ mice compared with the respective littermate controls (*n* = 16–25, four independent experiments). Data represented as mean ± SD.

In order to anticipate any potential role(s) for γδ T cells on testis steady-state physiology, we next investigated their localisation by microscopy by using γδ T-cell reporter mice (*TcrdH2BeGFP*). γδ T cells in *TcrdH2BeGFP* mice express a histone-bound eGFP; accordingly the expression of γδ T-cell-specific eGFP is very stable and located in the nucleus of γδ T cells. Ex vivo two-photon laser- scanning microscopy of testes extracted from adult *TcrdH2BeGFP* reporter mice and immobilised in an oxygen-flushed chamber revealed that most γδ T cells in the testis were motile (Fig. 4a; Supplementary Movie S1). Next, immunofluorescence microscopy of testis organ slices from *TcrdH2BeGFP* mice confirmed that γδ T cells were mainly found in the interstitial stromal compartment that surrounds seminiferous tubules (Fig. 4b). Thus, γδ T cells are in proximity to Leydig and Sertoli cells, which are responsible for testosterone production and spermatogenesis, respectively. Notably, both of these specialised testicular cell types are seemingly equipped to respond to IL-17 as shown by their receptor expression (Supplementary Fig. S3B). We therefore search for a potential impact of γδ17 cells on the biological functions of Leydig and Sertoli cells.

We first dissected the spermatogenesis process from WT, *Tcrδ*^−*/–*^ and *Il17*^*−/−*^ mice by flow cytometry, identifying the different germ cell stages according to their DNA content. No differences were found between *Tcrδ*^−*/−*^ and *Il17*^*−/−*^ mice and WT controls, indicating that testicular IL-17 does not impact on germ cell differentiation (Fig. 4c). Furthermore, *Tcrδ*^*−/−*^ and *Il17*^−*/*−^ mice did not show particular abnormalities of their overall testis morphology and histological structures (Fig. 4d). Finally, testosterone levels in the serum of *Il17*^−*/−*^ and *Tcrδ*^−*/*−^ mice were similar to their respective littermate controls (Fig. 4e). Altogether, these data support the absence of γδ T-cell overall impact on steady-state testis physiology, and go in line with a normal fertility displayed by our *Tcrδ*^*−/−*^ and *Il17*^*–/−*^ colonies.

### Testicular γδ17 cells promote immune surveillance against *Listeria monocytogenes*

Finally, as γδ T cells are well known to mediate immune surveillance in mucosal tissues as well as in the skin,^7^ we hypothesised that surveillance mechanism might be also important during testis infection. This concept has previously been suggested by studies, where depletion of γδ T cells exacerbated testicular inflammation upon bacterial infection.^12,33^ However, molecular mechanisms and pathophysiological outcomes have not been elucidated.

**Fig. 5:**
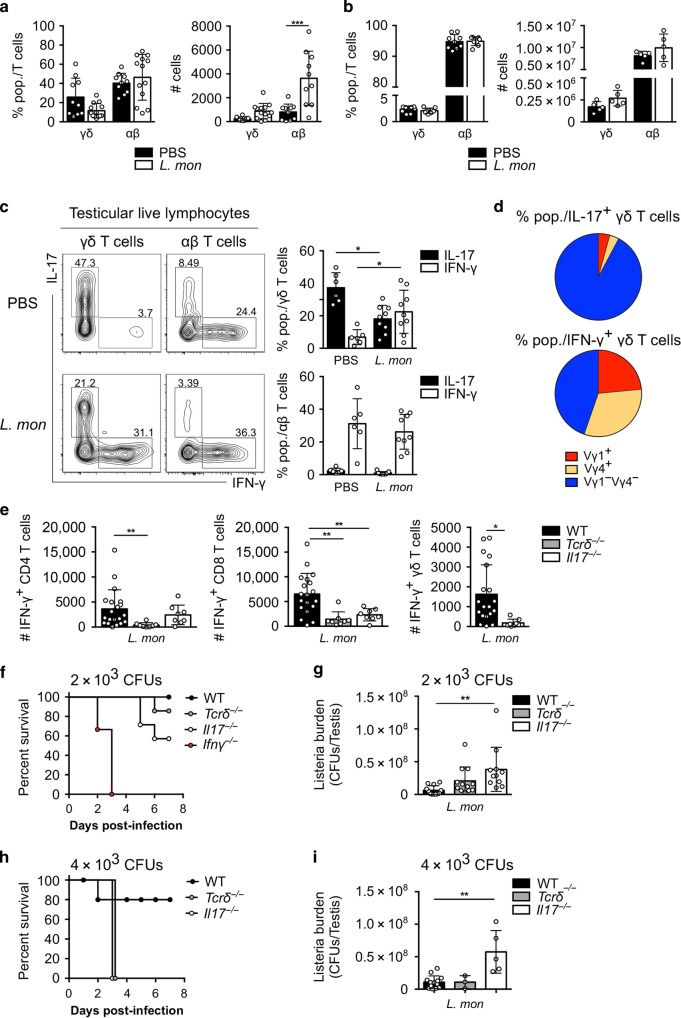
Testicular γδ17 promotes testis surveillance against *Listeria monocytogenes*. **a**, **b** Scatter plots show frequencies and numbers of γδ and αβ T cells among CD3^+^CD45^+^ cells after intra-testicular infection with *L. monocytogenes* (*L. mon*) (2 × 10^3^ CFU) (white) or PBS (black) in mature testis (*n* = 10–13, three independent experiments) (**a**) and spleen (*n* = 5–8, two independent experiments) (**b**). **c** Representative contour plots depicting IL-17^+^ or IFN-γ^+^ γδ (left) and αβ (right) T cells 3 days after intra-testicular injection of PBS (top) or *L. monocytogenes* (bottom). Scatter plots show frequencies of IFN-γ^+^ (white) and IL-17^+^ (black) γδ (top) and αβ (bottom) T cells (*n* = 10–13, three independent experiments). **d** Pie charts displaying the Vγ1^+^, Vγ4^+^ and Vγ1^–^Vγ4^–^ usage of IL-17^+^ γδ (top) and IFN-γ^+^ (bottom) γδ T cells 3 days after intra-testicular injection of *L. mon* (*n* = 8, two independent experiments). **e** Scatter plots show numbers of IFN-γ^+^ CD4 (left), CD8 (middle) and γδ (right) T cells in mature testis of WT (black), *Tcrδ*^−*/−*^ (grey) and *Il17*^*−/−*^ (white) mice after intra-testicular infection with *L. monocytogenes* (2 × 10^3^ CFU) or PBS (*n* = 8–19, three independent experiments). **f** Survival curve of WT (white), *Ifnγ*^*−/−*^ (red), *Il17*^*−/*−^ (white) and *Tcrδ*^−*/−*^ (grey) mice after intra-testicular injection of *L. monocytogenes* (2 × 10^3^ CFU) (*n* = 6–7, three independent experiments). **g** Bacterial burden (CFU per testis) of WT (black), *Tcrδ*^−*/−*^ (grey) and *Il17*^−*/−*^ (white) mice analysed 72 h after intra-testicular injection of *L. mon* (2 × 10^3^ CFU) (*n* = 11–12, three independent experiments). **h** Survival curve of WT (white), *Il17*^*−/−*^ (black) and *Tcrδ*^−*/−*^ (grey) mice after intra-testicular injection of *L. monocytogenes* (4 × 10^3^ CFU) (*n* = 5–7). Data pooled are represented as mean ± SD as evaluated by Kruskal–Wallis test followed by Dunn’s multiple-comparison test or one-way ANOVA followed by Holm–Sidak’s multiple-comparison test. *P < 0.05, **P < 0.01. **i** Bacterial burden (CFU per testis) of WT (black), *Tcrδ*^*–/–*^ (grey) and *Il17*^*−/−*^ (white) mice analysed 48 h after intra-testicular injection of *L. monocytogenes* (4 × 10^3^ CFU) (*n* = 3–5 animals/group). When possible, *Tcrδ*^*+/+*^ and *Il17*^*+/+*^ littermate controls were used and referred as WT animals.

Here we performed intra-testicular bacterial infection with *Listeria monocytogenes* (*L. monocytogenes*) as a model of experimental orchitis.^12^ We found that both γδ and αβ T cells accumulated in the infected testis (Fig. 5a)—but not in the spleen (Fig. 5b)—and mainly expressed IFN-γ (Fig. 5c, d). While the γδ17 cell Vγ-chain repertoire was mostly conserved upon infection compared with steady state, we observed that the IFN-γ^+^ population rather displayed a diverse repertoire composed of Vγ1^+^, Vγ4^+^ and Vγ1^–^Vγ4^–^ cells (Fig. 5d). Interestingly, the accumulation of testicular IFN-γ-producing γδ T cells after infection with *L. monocytogenes* was reduced in *Il17*^−*/−*^ mice compared with their WT littermate controls. Similarly, we observed a reduction of IFN-γ- producing CD8^+^ and CD4^+^ T cells in infected testes in *Il17*^*−/−*^ as well as *Tcrδ*^*–/−*^ mice, suggesting a role for γδ T cells and IL-17 in the amplification of the type 1 response in this model (Fig. 5e). This presence of IFN-γ producers was necessary to resolve intra-testicular bacterial infection, as all *Ifnγ*^*−/−*^ animals died within 3 days in response to a low (2 × 10^3^ CFUs) dose *of L. monocytogenes*, whereas 60% of *Il17*^−*/−*^, 80% of *Tcrδ*^*−/−*^ and 100% of WT mice were still alive at 8 days after infection (Fig. 5f).

This notwithstanding, testicular IL-17^+^ cells were required to control bacterial burden, as *Il17*^−*/−*^ displayed higher *L. monocytogenes* loads compared with their WT littermate controls 3 days post infection (Fig. 5g). Importantly, this translated into a poor survival outcome in response to a high (4 × 10^3^ CFUs) dose of *L. monocytogenes*, as all *Il17*^*−/−*^ and *Tcrδ*^*−/−*^ animals died within 3 days of infection, whereas 80% of the WT control mice were still alive after 8 days of infection (Fig. 5h). Interestingly, despite the higher susceptibility of *Il17*^−*/−*^ and *Tcrδ*^*−/−*^ animals after low- and high-dose infection, a compensatory expansion of other IL-17-producing lymphocytes, such as NKT and MAIT cells,^34^ (C. Paget, Personal communication) might have contributed to a lower bacterial burden in *Tcrδ*^*−/−*^ animals (Fig. 5i).

Altogether, our data highlight a crucial role for testicular γδ17 cells as a first line of defence against bacterial infection.

## Discussion

Over the past few years, tissue-resident γδ T cells have been explored in many specific tissues, such as the skin,^35^ lung,^36^ gingiva,^29^ eye,^37^ trachea^38^ and brain meninges,^21^ and also in the female sexual organs such as the uterus and vagina.^8,10,39^ Therefore, it is not surprising that γδ T cells are also present in the male reproductive organs. Unexpectedly, γδ T cells accounted for almost 50% of all lymphocytes in “immune privileged” testis. These testicular γδ T cells constituted a rather uniform population as almost all cells showed a terminally differentiated CD69^+^CD44^hi^CD62L^low^ phenotype, and their TCR repertoire was highly biased for Vγ6-containing rearrangements, which are known to take place during foetal life.^16^ This γδ T-cell phenotype appears to be unique to the testis, as other sex organs of the male reproductive tract, like the accessory glands, exhibit a diverse and heterogeneous population of γδ T cells implying the existence of a specialised immune cell network in immune-privileged testis compared with accessory glands.

In line with their CD44^hi^CD27^–^RORγt^+^ phenotype, we found that testicular γδ T cells produce IL-17 after ex vivo stimulation, and are in fact the main producers of this pro-inflammatory cytokine among all lymphocytes, whereas αβ T cells almost mostly secrete IFN-γ in the testis. Importantly, we observed a so-far-unknown expansion of murine testicular IL-17-producing Vγ6^+^ γδ T cells during puberty, around the age of 5 and 7 weeks. This dramatic accumulation of a cell population known to develop exclusively in the embryonic period^16^ led us to investigate the underlying mechanisms. At puberty, the testicular microenvironment is defined by specific changes in tissue structure, sexual hormones and microbiome composition.^23^ We showed that testicular γδ T cells depend on microbial signals and the TLR4–MYD88–IL-1α/IL-23 signalling axis. Interestingly, in contrast with the prominent role of IL-1β in the induction of IL-17 production by γδ T cells in the inflamed CNS,^32^ we show that testicular γδ17 cell homoeostasis at steady state is dependent on IL-1α. IL-1α is mainly produced by Sertoli and germ cells, and was previously shown to promote growth of immature Sertoli and spermatogonia cells,^40,41^ to inhibit Leydig cell steroidogenesis^42^ and to regulate blood–testis barrier dynamics by affecting actin skeleton of Sertoli cells.^43^ Thus, our work adds an immune-regulatory function of IL-1α within the testicular interstitial space, which is seemingly dependent on the microbiota.

Consistently, microbial cues have been previously reported to regulate γδ17 cell homoeostasis in other tissues, including the gut,^44^ skin,^35^ lung,^36,45^ liver^28^ and gingiva.^29^ Moreover, it was recently proposed that bacteria can also reside in organs assumed to be sterile and immune-privileged, such as the retina, where they promote IL-17 production by γδ T cells.^37^ Hence, it is conceivable that the testis itself could also harbour a resident microbiota that could modulate and shape testicular immune responses. Further investigations will be required to test this hypothesis.

On the other hand, it would be very interesting to identify the initial time point when foetal-derived γδ T cells migrate to the testis as well as molecules or stimuli required for the accumulation/expansion of these cells upon puberty.^16^ However, in this study, we observed that even in the absence of germs, TLR4, MYD88, IL-1R or IL-23R, a small population of Vγ6^+^ or γδ17 cells is still present in the testis. Therefore, we speculated that the initial development, but not the accumulation/expansion within the tissue, depends on microbial stimuli. Previously, it was shown that CCR6 promotes the migration of Vγ6^+^ γδ T cells to tissues at steady state.^22^ However, CCR6 was only expressed by a small fraction of testicular γδ17 cells, suggesting that other chemokines could be involved in their recruitment, while we also cannot formally exclude a downregulation of CCR6 expression after driving migration. Besides recruitment, in situ proliferation could thus also contribute to the increase in γδ T-cell numbers upon puberty. In line with this, we observed that Vγ6^+^ γδ T cells strongly proliferated in the pubertal testis, as mainly all of these cells expressed the proliferation marker Ki67. This substantial population then persisted throughout adulthood. In accordance, we found a reduced proliferative capacity of this cell subset after puberty and, moreover, tissue-resident Vγ6^+^ γδ T cells are characterised by their longevity.^46^

So what might be the physiological function of γδ17 cells in the testis? In vivo imaging revealed their motile, tissue-screening behaviour, which points to a role in tissue surveillance. Furthermore, we speculated that innate-like γδ T cells might be especially important for the fast–immune response against invading pathogens within the testis. The function of IL-17-secreting γδ T cells is already described in other immune-privileged sites, where they are associated with enhanced disease severity in stroke and experimental autoimmune encephalitis (EAE) within the brain,^47,48^ or increased protection against bacterial and fungal infection in the eye.^37^ Hence, rapid IL-17 production within the testis might be similarly important for immune defense against infection with bacteria.^49^ Interestingly, a unilateral intra-testicular inoculation of *L. monocytogenes* was shown to induce autoimmune-induced inflammation in the contralateral testis without spread of bacteria.^11^ While αβ T cells and macrophages play a detrimental role in the infected testis, Vγ6^+^ γδ T cells were shown to protect both infected and non-infected testis by producing IFN-γ, IL-2, IL-10 and TGF-β.^11,12^ Accordingly, we observed that intra-testicular infection with *L. monocytogenes* led to an infiltration of αβ and γδ T cells, in particular of IFN-γ-producing cells. This goes in line with previous data demonstrating that IFN-γ produced by neutrophils, NK or T cells upon *L. monocytogenes* infection promotes macrophage recruitment and consequently bacterial clearance and tissue.^50^ This crucial role of IFN-γ in the initial phase of *L. monocytogenes* infection is clearly demonstrated by the poor survival of *Ifnγ*^*−/*−^ mice. On the other hand, *Tcrδ*^*−/−*^ and *Il17*^*−/*−^ mice survived to the same dose of infection although displaying a higher bacterial load in the testis compared with WT controls. Interestingly, and in agreement with a previous study on the female reproductive tract,^51^ our results suggest that γδ17 cells boost the production of IFN-γ in situ and/or the recruitment of IFN-γ-producing subsets. Therefore, IL-17 seemingly promotes an amplification loop of the inflammatory response upon infection, as previously reported in EAE.^52^ Importantly, we observed very high susceptibility of *Il17*^*−/−*^ and *Tcrδ*^*–/−*^ mice to high (4 × 10^3^ CFU) doses of *L. monocytogenes*, which firmly establishes the protective role of mucosal γδ17 cells against bacterial infection in situ. Along the same line, recent findings have reported a protective role for Vγ6^+^ γδ T cells against *Candida albicans* in the female reproductive tract.^39^ Together, these examples may serve as a proof of concept for an antimicrobial immune surveillance of mucosal γδ17 cells in the male and female reproductive organs, predicting a similar response to other pathogens, such as *Escherichia coli (E. coli)* or *Chlamydia*, although further investigation will be needed to test this hypothesis.

In sum, we demonstrate that γδ17 cells are part of the immune system of the testis at steady state; they expand at puberty and make an important contribution to local tissue immune surveillance.

## Methods

### Mice

C57BL/6, *TcrdH2BeGFP*, *B6-Trcd*^*tm1Mal*^*Rag1*^*tm1.1Sadu*^ *Gt(ROSA)26Sor*^*tm1 (creERT2)Tyj*^ (here: *Indu-Rag1×TcrdH2BeGFP*),^53^ *C57BL/6-Il23r*^*tm1Kuch*^ (here *Il23r*^*gfp/gfp*^ or *Il23r*^*gfp/+*^), *Il23r*^*−/−*^, *Il1r*^*−/−*^, *Il1β*^−*/−*^, *Il1α*^−*/−*^, *Myd88*^*−/−*^, *Tlr2*^*–/−*^ and *Tlr4*^−*/*−^, *Tcrδ*^−/−^ and *Il17a*^*–/–*^ (referred as *Il17*^*−/−*^) and *Il17a*^*eGFP*^ × *Il22*^*sgBFP*^ reporter^18^ mice were kept in the Central Animal Facility at Hannover Medical School or Instituto de Medicina Molecular—João Lobo Antunes. When possible, *Tcrδ*^*−/*−^ and *Il17*^*−/−*^ mice were compared with their littermate controls that were co-housed from birth until weaning. WT germ-free (GF) mice were maintained in the Central Animal Facility at Hannover Medical School or in the GF facility of IGC (Oeiras, Portugal). Animals were purchased from Charles River or from the Jackson Laboratory. The cages are individually ventilated, so the animals were maintained under specific pathogen-free conditions. All experiments were approved by the animal ethics committee at the institutes and performed according to national and European regulations. For experiments 3–14-week-old males were sacrificed by CO_2_ inhalation and cervical dislocation. Testes, prostates and seminal vesicles were harvested after opening the abdomen from male mice.

### Haematoxylin and eosin staining

Frozen sections (5 µm) of mouse testis, prostate and seminal vesicle were fixed in ice-cold acetone for 10 min. The sections were stained with haematoxylin for 10 min and eosin for 30 s. After washing, slides were analysed by bright-field microscopy using a motorised upright Olympus BX61 fluorescence microscope with a ×10//0.4 objective (UPlanSApo, Olympus) and a F-View II camera (Olympus). Images were utilised by cellSens Dimension Software 1.12 (Olympus).

### Immunohistology

Frozen sections (7 µm) of mouse testis, prostate and seminal vesicle were fixed in ice-cold acetone for 10 min. After rehydration for 10 min and washing in TBST buffer, slides were blocked with 10% mouse serum diluted in TBST for 10 min. Next, the sections were incubated with diluted primary antibody anti-CD3 (Cy3, 17A2, in-house production with rat hybridoma cell lines), for 1 h at room temperature. After washing three times with TBST, the sections were stained either with diluted secondary antibody anti-rabbit IgG (Cy5, Jackson) for 45 min or with diluted DAPI for 3 min. The slides were washed two times with TBST. Sections were analysed by immunofluorescence microscopy using the previously described Olympus fluorescence microscope with Colour View IIIu camera (Olympus) and cellSens Dimension Software 1.12 (Olympus).

### Two-photon laser-scanning microscopy

For ex vivo imaging, extracted testes of *TcrdH2BeGFP* reporter mice were immobilised in an imaging chamber, which was flushed with oxygenated (95% O_2_/5% CO_2_) RPMI-1640 medium (Invitrogen) containing 1% penicillin/streptomycin, 25 mM HEPES and 5 g/litre glucose. The TriM Scope (LaVision BioTec) equipped with an upright Olympus BX51 microscope with a 203/0.95 water-immersion objective and a pulsed Ti sapphire-infrared laser (Mai Tai, SpectraPhysics) turned to 920 nm was used for imaging. The Imaris software 7.7.2 (Bitplane) was used for data analysis.

### Cell preparation for flow cytometry

Single-cell suspensions from testis, prostates and seminal vesicles were prepared by dissection of organs with a scalpel and incubation in 0.25 mg/ml collagenase D and 0.025 mg/ml DNase in RPMI-1640 medium with 10% FCS at 37 °C for 1 h. Digested solutions were passed through a 100-µm cell strainer, and lymphocytes were isolated by using a density-gradient centrifugation on 40% and 70% Percoll or Lympholyte. Cell suspensions were stained for flow-cytometry analysis using the following antibodies after blocking with 5% or 10% FC block: antibodies against CD45.2 (APCeF670, 104), CD44 (APC, IM7), CD3e (PECy7, 145-2C11), rat-IgM (PE, RM7B4), IL-17A (eFluor660, eBio17B7), CD69 (APC, H1.2F3), RORγt (APC, AFKJS-9) and T-bet (PE, eBio4B10) were purchased from eBioscience, antibodies against Tcrβ (PeCy7, H57-597), CD27 (PerCPCy5.5, LG.3A10), γδTCR (APC, GL3), Vγ1 (PE, 2.11), Vγ4 (PE, UC3-10A69), IL-17 (PE and PECy7, TC11-18H10.1), CD4 (BV605, RM-4.5) and CD8 (BV711, 53-6.7) were obtained from Biolegend, antibodies against CD45.2 (VioGreen, 104-2), CD3e (APCVio770 and VioBlue, 145-2C11), Tcrβ (APCVio700 and PerCPVio700, REA318), CD44 (VioBlue, IM7.8.1) and γδTCR (PEVio770, REA633) were purchased from Miltenyi, antibodies against CD3e (PE, 145-2C11), CCR6 (A647, 140706) Vγ5 (APC, 536) and ki67 (PECy7, B56) were obtained from BD Biosciences, antibody against IFN-γ (PECy7, XMG1.2) was ordered from Thermo Fisher Scientific and antibodies against Vγ4 (Cy5, 49-2.1), γδTCR (Alexa488, GL3) and 17D1 were produced in-house with rat hybridoma cell lines.

To check the viability, cells were stained either with Zombie Aqua Dead Cells or LiveDead Fixable Viability Dye (Invitrogen) before blocking or with DAPI after surface staining. Samples were acquired using LSRII (BD Biosciences) or FACSFortessa (BD Biosciences). Data were analysed using FlowJo software (Tree Star).

### Cytokine measurement

Before staining for intracellular cytokines, isolated single cells were stimulated in 96-well plates in RPMI-1640 medium (containing 1% glutamine (100×), 1% PenStrep and 10% FCS) with PMA (final concentration 50 ng/ml) and ionomycin (final concentration 2 µg/ml) and incubated for 3 h at 37 °C with Brefeldin A (final concentration 10 µg/ml). Cells were stained for surface molecules and then treated with Cytofix/Cytoperm according to the manufacturer’s protocol (BD Biosciences). Ultimately, cells were stained for intracellular IL-17 and IFN-γ.

### Spermatogenesis assay

Testicular preparations were isolated using a two-step enzymatic digestion to remove interstitial cells, as previously described.^54^ Briefly, the testis tunica albuginea was removed, and seminiferous tubules were dissociated by enzymatic digestion with 0.5 mg/ml of collagenase D (Roche) and 200 μg/ml DNAseI (Roche) for 20 min at 35 °C in complete DMEM: F12 (Invitrogen), supplemented with 1 mM L-glutamine, 5 mM sodium L-lactate, 1 mM sodium pyruvate and 0.1 mM MEM nonessential amino acids (all from Invitrogen Life Technologies). The suspension was layered over 5% Percoll gradient (GE Healthcare) and allowed to settle for 20 min. The bottom Percoll composed of interstitial cell was digested with 200 μg/ml DNaseI and 1 mg/ml trypsin for 20 min at 35 °C, and foetal bovine serum (FBS) was added to halt the digestion. The digested product was filtered through a 70-μm and a 40-μm cell strainer, washed in PBS and centrifuged at 500 × *g* for 10 min, and the resulting pellet was resuspended in complete DMEM:F12 supplemented with 5% FBS. Cell suspensions was stained with 7AAD for 1 h at 4 °C for analyses of the cell cycle. Testicular cell population was referred as 4 N (tetraploid testicular cells, premeiotic spermatocyte I), 2 N (diploid testicular cells) and N (haploid spermatids).

### Enzyme-linked immunosorbent assay (ELISA)

Levels of testosterone (R&D System) from serum and levels of IL-1α, IL-23 and IL-1β (Invitrogen) from the testis were measured by ELISA, according to the manufacturer’s instructions. Blood was removed from the heart, settled for 30 min at room temperature and centrifuged at 15,000 × *g* for 10 min at RT. Serum supernatant was collected and stored at −20 °C until used. Total protein content was quantified using the BioRad DC Protein Assay kit.

### RNA isolation, cDNA production and real-time PCR

For mRNA expression analysis, total RNA was extracted with the high pure RNA isolation kit (Roche), according to the manufacturer, from sorted γδ T cells, CD4 T cells, CD8 T cells, monocytes, dendritic cells, neutrophils or enriched Sertoli and Leydig cells, as previously described.^54^ RNA concentration and purity were determined using the NanoDrop^TM^ 2000 spectrophotometer (Thermo Fisher Scientific).

For mRNA, reverse transcription was performed with random oligonucleotides (Invitrogen) using Moloney murine leukemia virus reverse transcriptase (Promega). The total RNA was reverse-transcribed into cDNA using the T100® Thermal Cycler (BioRad), and all quantitative PCRs (qPCRs) were performed in MicroAmp® Optical 384-Well Reaction Plate (Applied Biosystems) using the RT-PCR ViiA7^TM^ system (Applied Biosystems). For mRNA expression analysis, primer sets (Sigma) designed by Universal Probe Library Assay Design Center (Roche) were used on the cDNA previously obtained, and relative quantification of specific cDNA species to endogenous references Beta-Actin or Beta2-microglobulin was carried out using SYBR on ViiA7 cycler (Applied Biosystems). Data were analysed using ViiA7^TM^ software v1.2.1.

### *Listeria monocytogenes* infection

Nine- to 12-week-old male mice were used for the experiments (*L. monocytogenes* strain EGD). Mice were inoculated into both testes under the tunica albuginea with 2 × 10^3^ or 4 × 10^3^ colony-forming units (CFU) of *L. monocytogenes* in 20 µL of PBS.

### Statistical analysis

Statistical analysis was performed using Graphpad Prism. The values presented are mean ± SD of *n* independent experiments. To test the significance of the differences between two conditions, a Student’s *t* test and Mann–Whitney were used. *P* values of <0.05 were considered to be statistically significant. Statistical analyses are described in more detail in figure legends.

## Supplementary information

Supplementary information

Supplementary Movie 1
