# Supplementary material for: Microbiota-dependent expansion of testicular IL-17-producing Vγ6+ γδ T cells upon puberty promotes local tissue immune surveillance
Source: Mucosal Immunol. 2020 Jul 30;14(1):242–52. doi: 10.1038/s41385-020-0330-6 (PMC7790758; doi:10.1038/s41385-020-0330-6)
Supplement: Supplementary file 1 — Supplementary information [file 41385_2020_330_MOESM1_ESM.pdf]

Sup. Figure 1

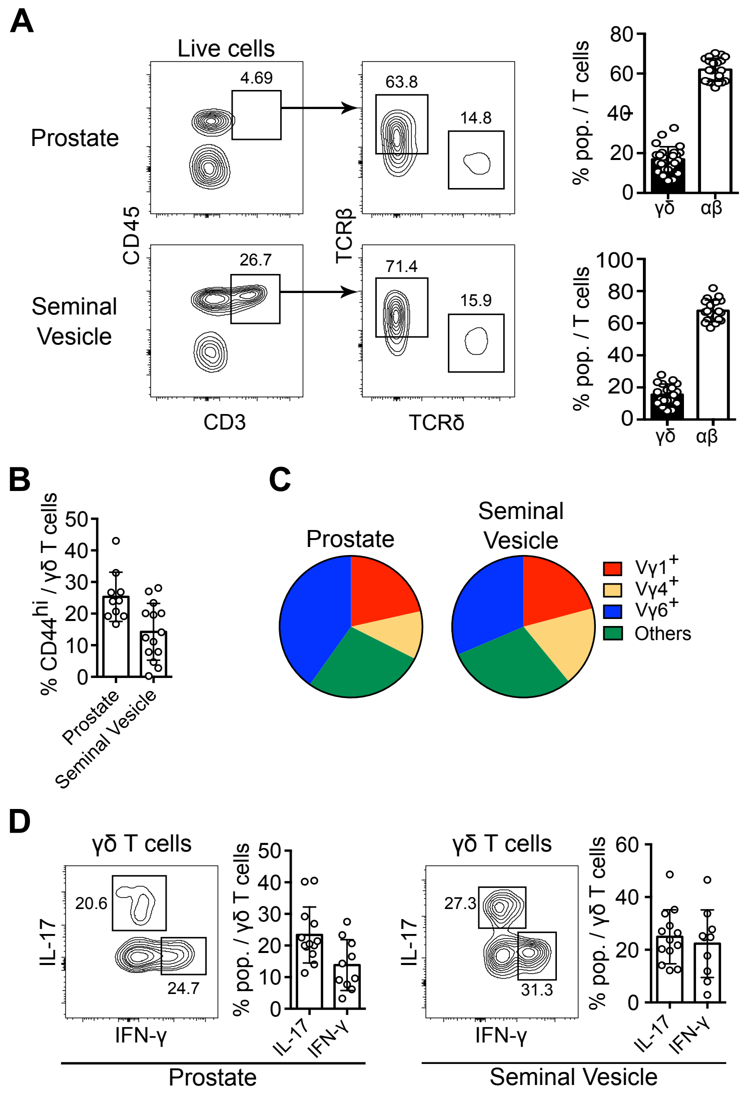

Figure S1. γδ T cells reside in the prostate and seminal vesicle with a diverse effector function. (A) Representative contour plots depicting γδ and αβ T cells (right) gated on $CD3^+CD45^+$ cells (left) in the prostate (top) and seminal vesicle (bottom). Scatter plots show frequencies of γδ (black) and αβ (white) T cells among $CD45^+CD3^+$ cells (n=19-26, 5 independent experiments). (B) Scatter plot displays frequencies of CD44 expression on γδ T cells in the prostate and seminal vesicle (n=10-14, 3 independent experiments). (C) Pie charts depicting mean frequencies of $V\gamma1^+$, $V\gamma4^+$, $V\gamma6^+$, and other γδ T cell subsets in prostate (left) and seminal vesicle (right) (n=10-11, 3 independent experiments). (E) Representative contour plots and scatter plots showing IL-17 and IFN-γ production among γδ T cells in the prostate (left) and seminal vesicle (right) (n=10-13, 3 independent experiments). Data are represented as mean ± SD.

Sup. Figure 2

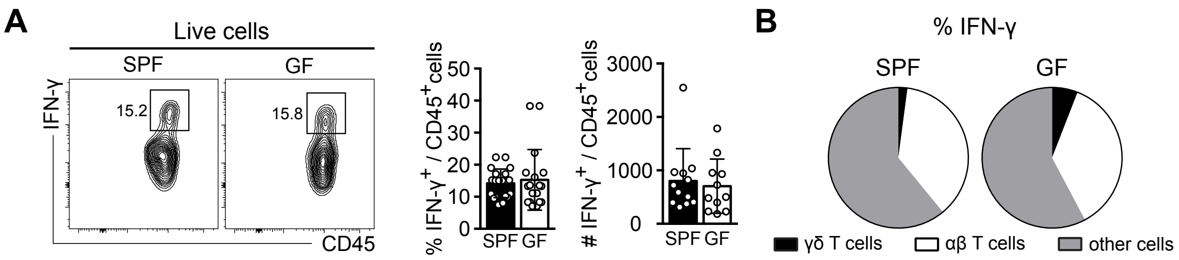

Figure S2. Testicular IFN-γ production is not dependent on microbiota signals. (A) Representative contour plots depicting IFN-γ production against CD3 expression gated on all $CD45^+$ cells in the testes of specific pathogen-free (SPF) (left) and germ-free (GF) (right) mice. Scatter plots show frequencies and numbers of IFN-γ^+ cells among $CD45^+$ cells (n=11-20, 3-5 independent experiments). (B) Pie chart depicting mean frequencies of immune cell subsets contributing to IFN-γ production in testes of SPF (left) and GF (right) mice (n=11-12, 3 independent experiments). Each dot represents one mouse. Data are pooled from 3-5 independent experiments and are represented as mean ± SD.

Sup. Figure 3

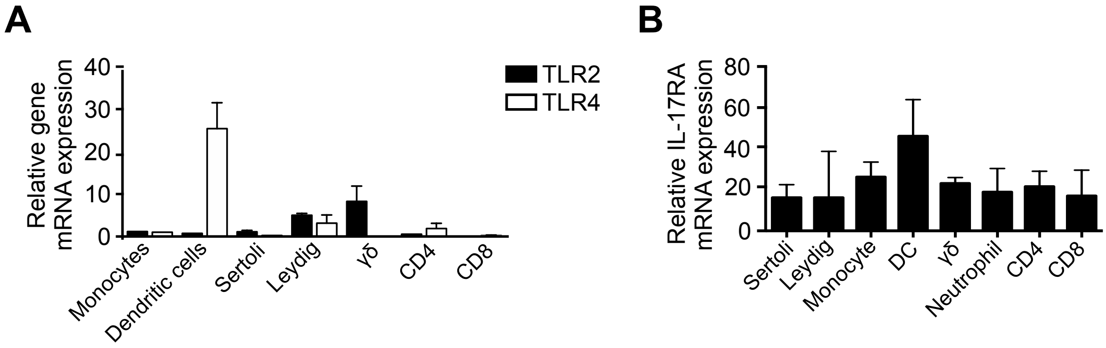

Figure S3. TLR2, TLR4 and IL-17RA expression in different testicular cell subset. (A) mRNA expression of TLR2 (black) or TLR4 (white) relative to b-actin and b2-microglobulin reference genes in the indicated testicular cell subsets (n=2-8, 1-2 independent experiments) (B) mRNA expression of IL-17RA relative to beta-actin and beta 2-microglobulin reference genes in the indicated testicular cell subsets (n=2-8, 1-2 independent experiments). Data is represented as mean ± SD.
